# Echocardiography screening of German military pilot applicants as an example for high-hazard occupations

**DOI:** 10.3389/fpubh.2025.1436805

**Published:** 2025-03-12

**Authors:** Norbert Guettler, Stefan Sammito

**Affiliations:** ^1^German Air Force Centre of Aerospace Medicine, Cologne, Germany; ^2^Department of Cardiology, University Hospital Giessen, Medical Clinic I, Justus Liebig University, Giessen, Germany; ^3^Department of Occupational Medicine, Medical Faculty, Otto von Guericke University, Magdeburg, Germany

**Keywords:** pre-employment screening, echocardiography, pilot applicant, high-risk occupation, aeromedical assessment, military

## Abstract

**Introduction:**

Pre-employment screening is of utmost importance in high-risk occupations for the early diagnosis and prevention of cardiac and non-cardiac disease, and for risk mitigation. Recommendations for echocardiography screening, however, are contradictory. It was the aim of this study to retrospectively analyze echocardiography data from German military pilot applicant screening to find out in how many cases cardiac disease was diagnosed, and how often the diagnosis influenced aeromedical decision making.

**Methods:**

6,110 screening echocardiographies from German military pilot applicants, 5,923 were male, examined between January 2007 and June 2020 were retrospectively analyzed for findings and their impact on aeromedical decisions.

**Results:**

During a 14.5-year period, 4,477 out of 6,110 screening echocardiograms were normal. The remaining 1,633 applicants revealed a total of 1,962 abnormalities, mainly consisting of minor tricuspid and mitral valve regurgitations (81.9%). Due to echocardiography findings, 80 applicants (1.3%) were unfit for flying duties, 9 (0.1%) were fit with limitations, and 1,544 (25.3%) were fit with findings that had to be monitored over time, but which were not aeromedically relevant. The most common diagnoses leading to disqualification or limitations were bicuspid aortic valve with or without aortic regurgitation (84.9%) and mitral valve prolapse with or without regurgitation (9.3%).

**Conclusion:**

Percentages of abnormal findings were similar to other studies. Aeromedical assessments based on those findings, however, were slightly different, as they depend on national employment policies. As a consequence, the usefulness of echocardiography may vary between different countries and different professions, depending on the acceptance of certain findings for employment.

## Introduction

High-hazard occupations require optimal health in the workforce to mitigate the risk of incapacitation and distraction and so to reduce safety risks to themselves, other employees, and the general public (1). These professions include pilots, astronauts, divers, mountaineers, offshore workers, emergency workers (firefighters, emergency healthcare workers, law enforcement personnel), and professional drivers. For example, there have been a number of aircraft incidents and accidents, some of them with fatalities, that were directly caused by myocardial infarctions of the pilots flying (2).

Pre-employment screening and periodic medical examinations in accordance with national and international laws and regulations are of utmost importance for those high-risk occupations for the early diagnosis and prevention of cardiac and non-cardiac disease, and for risk mitigation. The early recognition of health problems is especially important for first applicants, as they may spend their whole career in a high-risk profession. A premature end of their career because of a disease should be prevented as well as an aggravation of an undetected disease by the exercise of their profession.

Cardiovascular disease is one of the most common medical reasons for incapacitation or distraction of high-hazard employees (3, 4). Therefore, a variety of cardiovascular screening recommendations exists for different professions, e. g. regarding screening by electrocardiography or exercise electrocardiography (5–7). Recommendations for echocardiography screening, however, are contradictory, although the diagnosis of many cardiac diseases and abnormalities is not possible without echocardiography or more sophisticated cardiac imaging (8).

The air forces of some allied countries including the US Air Force do not perform echocardiography screening in pilot applicants, because the number of abnormal findings in the specific age group, most applicants are aged from 18 to 25 years, is low, and echocardiography screening is therefore deemed to be not efficacious (9). A different attitude is shared by the air forces of other countries including Germany, which routinely perform echocardiography screening in pilot applicants, based on the assumption that certain abnormal findings are of such importance for aeromedical assessment that echocardiography screening is justified.

Among the group of high-hazard occupations, pilots are very intensely regulated by a variety of medical standards issued by national and international organizations and licensing authorities (10). Pilot screening can therefore be used as an example for other high-risk occupations.

The aim of this study is to retrospectively analyze echocardiography data from German military pilot applicant screening in order to find out in how many cases cardiac disease has been diagnosed, and how often the diagnosis has influenced aeromedical decision making.

The study has to be seen in the context of an intended harmonization and possibly standardization of screening programs among allied countries. Recently, a North Atlantic Treaty Organization (NATO) working group (Human Factors and Medicine (HFM) 386) has been initiated as a three-year project, titled “Cardiac Screening on Military Aircrew and Other High Demand Military Personnel” and working on these questions.

## Materials and methods

The digital information system (DIL 2006, Noris Ingenieurbüro GmbH, Nürnberg, Germany) of the German Air Force Centre of Aerospace Medicine was searched for pilot applicant screening from January 1, 2007, to June 30, 2020. All echocardiography results were retrospectively analyzed, age, height, weight, and body mass index were also captured. If the digital documentation was not conclusive, the original paper documentation was screened for additional information. Most applicants were civilians, a minority was already part of the armed forces, air force, army, or navy, and was performing other duties. All applicants were pre-selected in an assessment center including a simple medical screening not including echocardiography. All applicants for flying duties independently of their future branch (air force, army, navy) or their future aircraft type (high-performance aircraft, fixed-wing aircraft, rotor-wing aircraft, drones) are screened in the German Air Force Centre of Aerospace Medicine for the German Armed Forces (Bundeswehr).

In total, 6,609 examinations were performed during the analyzed period. Out of these, 31 were excluded, because the same person was examined twice. In these cases, only the first screening echocardiography was included in the study. Six additional examinations were excluded, because the applicants were examined twice, and only in the second examination a screening echocardiography was carried out. Only this second examination was inlcuded in the study. So, in total, 6,572 examinations from different applicants could be used. Out of these, 444 examinations (6.8%) had to be excluded, because no echocardiography screening was performed. An additional 18 examinations (0.3%) were excluded, because the echocardiography diagnosis was not usable, as both the digital and the original paper documentation were not meaningful. This means that 6,110 echocardiograms could be included in this study (92.3% of all applicants screened during this period of time), 5,923 (96.9%) were males and 187 (3.1%) were females. The average age of the applicants was 21.1 ± 3.3 [95%-Confidence interval (CI): 21.0–21.2] years (min: 16.4 years, max. 52 years), the body height was 180.3 ± 6.7 cm (95%-CI: 180.1–180.5 cm; min: 155.1 cm, max: 204.0 cm), the body weight 75.7 ± 9.9 kg (95%-CI: 75.4–75.9 kg; min: 50.3 kg, max: 121.4 kg), and the body mass index (BMI) was 23.3 ± 2.5 kg/m^2^ (95-CI: 23.2–23.3 kg/m^2^; min: 16.7 kg/m^2^, max: 35.5 kg/m^2^).

Echocardiographies were performed with Vivid 7 and Vivid E9 by GE HealthCare Technologies, Inc., Chicago, IL, United States. All examinations were performed by physicians with special training in echocardiography. As minimum requirements the examinations consisted of two-dimensional echocardiography, color doppler, pulsed wave doppler, and continuous wave doppler. Three-dimensional echocardiography or tissue doppler were added on indication. Standard views were left parasternal views (long and short axis) and left apical views (four-chamber, five-chamber, two-chamber, three-chamber). Right parasternal and apical view, suprasternal, and subcostal views were added on indication. Assessments and measurements were performed in accordance with current guidelines and literature. Examinations were stored on the hard drive of the device and later on archived on Compact Disc Read-Only Memory (CD ROMs). Printouts of the results were archived in paper records; the results and measurements were transferred into the institute information system.

For descriptive statistic IBM SPSS Statistik 24 (IBM, Armonk, NY, United States) was used. Unless otherwise stated, the mean and standard deviation are given with the 95% confidence interval (95%-CI).

According to the regulations of the North Rhine Medical Association, Germany, the responsible authority for this study, a vote of the ethics committee was not necessary for this retrospective analysis.

## Results

During the analyzed 14.5-year period, 6,110 PMEs with screening echocardiographies were included (5,923 were males and 187 females). Out of these, 4,477 applicants had a normal echocardiogram (4,349 were males and 128 females, age was 21.1 ± 3.3 [21.0–21.2] years, BMI was 23.3 ± 2.6 [23.2–23.4] kg/m^2^). The remaining 1,633 applicants (1,574 were males and 59 females, age was 21.1 ± 3.3 [95%-CI: 20.9–21.2] years, BMI was 23.2 ± 2.5 [95%CI: 23.1–23.3] kg/m^2^) revealed a total of 1,962 different abnormalities. Most common were tricuspid valve regurgitation (in 61.9% of the 1,633 cases), mitral valve regurgitation (20.0%) and ballooning mitral valve leaflet (7.5%). An overview of all findings is listed in Table 1.

**Table 1:** Echocardiographic abnormalities.

Finding	*n*	%
Tricuspid regurgitation	1,029	61.9
Mitral regurgitation	332	20.0
Ballooning mitral leaflet	124	7.5
Pulmonary regurgitation	120	7.2
Aortic regurgitation	88	5.3
Bicuspid aortic valve	73	4.4
Hypermobile interatrial septum	36	2.2
Left ventricular enlargement	24	1.4
Patent foramen ovale	21	1.3
Interatrial septal aneurysm	20	1.2
Chiari network	13	0.8
Mitral valve prolapse	13	0.8
Hyperkinetic heart	11	0.7
Myxomatous mitral valve	11	0.7
Left ventricular hypertrophy	8	0.5
Left atrial enlargement	7	0.4
Diastolic dysfunction	5	0.3
Ectasia of the ascending aorta	5	0.3
Elevated pulmonary artery pressure	4	0.2
Hypermobile chorda of the mitral valve	4	0.2
Right ventricular enlargement	4	0.2
Atrial septal defect	1	0.1
Asynchronous left ventricular function	1	0.1
Reduced left ventricular ejection fraction	1	0.1
Reduced right ventricular ejection fraction	1	0.1
Annuloaortic ectasia	1	0.1
Interatrial thrombus	1	0.1
Aortic valve sclerosis	1	0.1
Right atrial enlargement	1	0.1
Ventricular septal defect	1	0.1
Status after cardiac surgery	1	0.1

One person could have more than one finding.

Due to echocardiography findings, 77 applicants (1.26%) were assessed as unfit for flying duties, 9 (0.15%) were fit with limitations, and 1,544 (25.3%) were fit with findings that had to be monitored over time, but which were currently not aeromedically relevant. Tables 2, 3 list findings of applicants who were unfit for flying duties or fit with limitations, respectively.

**Table 2:** Echocardiographic abnormalities in applicants who were unfit for flying duties.

Finding	*n*	%
Bicuspid aortic valve	56	70.0
Bicuspid aortic valve plus aortic regurgitation	15	18.8
Aortic regurgitation	2	2.5
Ectasia of the ascending aorta	1	1.3
Mitral and pulmonary regurgitation	1	1.3
Bicuspid aortic valve plus aortic regurgitation and mitral valve prolapse	1	1.3
Bicuspid aortic valve plus aortic regurgitation and ectasia of the ascending aorta	1	1.3

**Table 3:** Echocardiographic abnormalities of applicants who were fit for flying duties with limitations.

Finding	*n*	%
Mitral valve prolapse	7	77.8
Atrial septal defect	1	11.1
Pulmonary regurgitation	1	11.1

## Discussion

Out of 6,110 applicants within the analyzed 14.5-year period 1.26% were assessed as unfit, 0.15% were fit with limitations due to their echocardiographic findings. The most common diagnoses leading to disqualification or limitations were bicuspid aortic valve (BAV) with or without aortic regurgitation and/or dilatation of the ascending aorta (84.9, 1.2% of all applicants) and mitral valve prolapse (MVP) with or without regurgitation (9.3, 0.13% of all applicants). Although the number of abnormalities leading to disqualification or limitations was small, detection is important in the light of the challenging working environment of military pilots and a whole career of mostly several decades lying ahead of them.

In other studies, in this field, the rate of aeromedically relevant echocardiographic findings has been reported similar with up to 3.31% in the scientific literature (8, 9, 11). Depending on national aeromedical policies the disqualification rate could be lower (8, 9). So, an analysis of echocardiographic screening examinations by the United States Air Force School of Aerospace Medicine with 20,208 pilot applicants included shows a similar percentage of diagnoses (9). The disqualification rate, however, was much lower in that study especially for BAV, leading to the conclusion that screening echocardiography for military pilot applicants was deemed not efficacious.

Another retrospective study of 2,657 Israeli Air Force candidats found echocardiographical findings affecting aeromedical decisions in 3.31% of all candidates (8). They resulted in disqualification of 0.94% of all candidates, limitation to low-performance aircrafts in 0.83%, and need for follow-up in additional 1.54%. Another Israeli study analyzed 7,042 routine echocardiographies in air force academy applicants (11) specifically for BAV. BAV was found in 1.35% of those applicants, in 36% BAV was associated with mostly mild aortic regurgitation.

Echocardiography screening is also discussed in competitive athletes to exclude cardiac disease with an increased risk of sudden cardiac death (SCD) including cardiomyopathies (12–18). The authors of a survey across European Society of Cardiology member countries, however, conclude that in the absence of scientific evidence, before such practice is recommended, large studies using echocardiography in the pre-participation evaluation setting are necessary (19).

A major finding in our study and also in the analysis of Grossman et al. (11) was the number of BAV. This is similar to other prevalence studies in the normal population, in which the prevalence of BAV is 0.5 to 2% in the United States (20). The valvular pathology commonly seen in BAV includes aortic valve stenosis, regurgitation, and infective endocarditis. The findings by Grossman et al. revealed that the cardiac morphology of young healthy individuals with BAV was affected irrespective of the presence or absence of mild or moderate aortic regurgitation. It was therefore recommended that aeromedical decisions should be based on the finding of BAV, and not on the presence of an associated aortic regurgitation (11). Because BAV confers an estimated 50% lifetime risk of requiring valve replacement and the procedures required for BAV management account for more morbidity and mortality than all other congenital heart diseases combined (20), it leads to disqualification for high-hazard occupations including flying a military aircraft in accordance with common regulations (21, 22). In the USAF, BAV is also disqualifying, but a waiver can be granted for uncomplicated BAV.

In the German Armed Forces, applicants with BAV are usually unfit for flying duties, and also unfit for military service according to the German regulations (21, 23). The acceptable means of compliance issued by the European Union Aviation Safety Agency allows for fit assessment of professional pilot (class 1) applicants with BAV after referral to the licensing authority as long as no other cardiac or aortic abnormality is demonstrated (24). One reason why the military regulations in Germany are more stringent than the European civilian regulations is the exacting working environment of military pilots, especially for those flying high-performance aircraft, including acceleration, hypobaric hypoxia, tactical flying maneuvers, missions in austere environments, and possible enemy action (9). Another reason is the fact that in case of an employment, military pilot careers usually last for two to four decades during which an aggravation of the disease by their profession should be avoided. Overall, most individuals with BAV will develop valvular and/or aortic complications related to their BAV (20). As the fitness decision not only for military pilots but for military personnel at large is a long-term decision affecting the whole military career, the risk of employing those individuals is deemed too high by many armed forces, including the German Armed Forces.

The prevalence of mitral valve prolapse (MVP) is 1.3 to 3% in the general population (25). MVP alone is not necessarily disqualifying in the German Armed Forces but may lead to an exclusion from high-performance flying. Those associated with relevant mitral regurgitation, arrhythmia, bileaflet prolapse, thickened mitral leaflets, or mitral annulus disjunction are usually assessed as unfit for flying duties. Overall, MVP is a benign disease in most cases, associated with non-specific symptoms, such as atypical chest pain, exertional dyspnea, palpitations, anxiety, mid-systolic click, low blood pressure, and leaner build, which have come to be known as MVP syndrome. MVP can also be associated with electrocardiographic abnormalities and complex ventricular arrhythmias with polymorphic/right bundle branch block morphology. The complications associated with valve disease include mitral regurgitation and less common infective endocarditis and cerebrovascular ischemic events. A subset of patients can experience cardiac arrest or SCD due to complex ventricular arrhythmias, which is a dramatic event that can affect these otherwise young healthy subjects (26). The incidence of SCD is estimated to be 0.14–1.8% per year in patients with MVP (27) with a prevalence of 2.3% (28), but it was reported to be as high as 7% in a young SCD population (13% in the female population) (29). The estimated rate of SCD in the general population is 0.06–0.08%/year, but the risk of SCD in MVP and cardiac patients is 1.73–2.3 times higher.

Screening echocardiography regularly reveals abnormal findings in young apparently healthy people. The most common ones are BAV with a prevalence of 0.5 to 2% (20) and MVP with a prevalence of 1.2 to 3% (25). Mild or moderate regurgitations of heart valves without morphologic changes are also common. The consequences of those findings, however, are a policy decision by the employer. If the prognosis and the likelihood of complications during a whole career is considered, the findings may more often lead to disqualification than if a fitness decision only relies on the current condition. The question if routine echocardiography for young and apparently healthy individuals should be implemented in applicant screening depends on such considerations for decision making. This is not only valid for pilots or non-pilot aircrew, but also for other high-hazard occupations including divers, astronauts, law-enforcement personnel, paramedics, off-shore workers, or professional drivers.

Aiming at harmonization or even standardization of air force screening programmes for pilot applicants, not only the prevalenve of certain findings is important, but also the effect of certain findings on aeromedical decisions. While our study for example revealed that the percentage of BAV was similar to that in the US and other countries, the consequences are different, as in Germany BAV is not compatible with flying, while at least an uncomplicated BAV (without relevant regurgitation, stenosis, or infection) can be waivered in the US. Therefore, a screening without echocardiography can more easily be justified in the US than in Germany, although a small risk of missing a BAV with aortic valve disease or other relevant findings has to be accepted.

Such retrospective prevalence studies in the specific age group of military pilot applicants is an important first step towards the harmonization of screening policies among allied countries and the improvement of international military cooperation. Perhaps aeromedical regulations and waiver guides will also have to be aligned over time. The results obtained in our study and in the whole project can also be applied to other high-hazard occupations. The recently initiated three-year effort of the NATO working group HFM-386 will further elaborate these potentials.

Our study has some strengths and limitations, which have to be considered. One strength is that we could include all examinations for pilot applicants over the 14.5-year-period, because there is only one aeromedical examination center for the German Armed Forces, and because the German Air Force Centre of Aerospace Medicine used a digital documentation system. On the other hand, echocardiographies were performed by many different physicians during that timeframe. Therefore, a certain interobserver variability cannot be excluded. In addition, policies for aeromedical assessment might have slightly changed during that period of time as well as guidelines and recommentations for echocardiography in general. To reduce the effect of such changes, every examination was reevaluated regarding aeromedical fitness during our analysis.

It should also be recognized, that applicants for military aircrew duty receive a first screening in an assessment center based on medical history and a standardized questionnaire, before a profound aeromedical screening including echocardiography is performed. Thus, applicants with known heart disease are already classified as unfit during their basic screening. This means that the study population had been classified as a healthy population without any known (heart) disease as a result of their basic screening in the assessment center. The findings reported in our study have to be discussed against this background. Because our findings are similar to those of other studies (8, 9, 11), we think the bias caused by this effect is small for our cohort.

It should also be recognized, that the number of women examined here was low with only 3.1%. This was caused by a low overall proportion of women in the German Armed Forces of approximately 10%. An equal proportion of men and women could therefore not be expected in this cohort study, especially not in the group of pilots, where the percentage of women is much lower than in the whole German Armed Forces.

In summary, the percentage of abnormal findings in our study was similar to other studies and to that reported in the literature. Aeromedical assessments based on those findings, however, were slightly different and depend on national employment policies. As a consequence, the usefulness of echocardiography may vary between countries and different professions, depending on the acceptance of certain findings for employment. This and other studies aim at a harmonization of screening policies for pilot applicants and other high-risk occupations.

## Data Availability

The data analyzed in this study is subject to the following licenses/restrictions: analyzed datasets are property of the German Armed Forces (Bundeswehr). They are available upon reasonable request. Requests to access these datasets should be directed to Norbert Guettler, norbertguettler@bundeswehr.org.
